# Overcoming challenges in prevalence meta-analysis: the case for the Freeman-Tukey transform

**DOI:** 10.1186/s12874-025-02527-z

**Published:** 2025-04-05

**Authors:** Jazeel Abdulmajeed, Tawanda Chivese, Suhail A. R. Doi

**Affiliations:** 1 Department of Population Medicine, College of Medicine, QU Health, Qatar University, Doha, Qatar; 2 Division of Sciences and Mathematics, School of Interdisciplinary Arts and Sciences, University of Washington Tacoma, Tacoma, WA USA

**Keywords:** Meta-analysis, Prevalence, Transforms, Logit transformation, Freeman-Tukey transformation

## Abstract

**Background:**

Traditional statistical methods assume normally distributed continuous variables, making them unsuitable for analysis of prevalence proportions. To address this problem, two commonly utilized variance-stabilizing transformations (logit and Freeman-Tukey) are empirically evaluated in this study to provide clarity on the optimal choice among these transforms for researchers.

**Methods:**

Simulated datasets were created using multiple Monte Carlo simulations, with varying input parameters to examine transformation estimator performance under varying scenarios. Additionally, the research delved into how sample size and proportion influenced the variability of the Freeman-Tukey transform. Performance was evaluated for both single prevalence proportions (coverage, interval width and variation over sample size) as well as for meta-analysis of prevalence (absolute mean deviation of pooled proportions, coverage and interval width).

**Results:**

For extreme proportions we found that the Freeman-Tukey transform provides better coverage and narrower intervals compared to the logit transformation, and for non-extreme proportions, both transformations demonstrated similar performance in terms of single proportions. The variability of Freeman-Tukey transformed proportions with sample size is only seen when the range of proportions under scrutiny are very small (~ 0.005), and the variability of the Freeman-Tukey transform’s value occurs in the third decimal place (0.007). In meta-analysis, the Freeman-Tukey transformation consistently showed lower absolute deviation from the population parameter, with narrower confidence intervals, and improved coverage compared to the same meta-analyses using the logit transformation.

**Conclusion:**

The results suggest that the Freeman-Tukey transform is to be preferred over the logit transformation in the meta-analysis of prevalence.

**Supplementary Information:**

The online version contains supplementary material available at 10.1186/s12874-025-02527-z.

## Background

The findings from many conventional epidemiological studies are presented in the form of prevalence proportions playing a fundamental role in clinical research. These prevalence proportions are typically bound within the 0 and 1 range and therefore analyzing prevalence data presents unique challenges since traditional statistical methods assume normally distributed continuous variables [1]. Prevalence proportions also exhibit heteroscedasticity, with variance typically increasing towards the boundaries of 0 and 1 [1]. To address these issues, statistical methods make use of variance-stabilizing transformations of prevalence proportions and results are then back-transformed for interpretation. Such transformed prevalence proportions aid in better approximations to the normal distribution while enabling meta-analytic pooled proportions to keep to the 0 or 1 limits. Also, since they are unconstrained to the 0 to 1 range, transformed proportions avoid the squeezing of variance observed at extremes of natural proportions [1]. The two most commonly used variance-stabilizing transformations are the logit transformation and the Freeman–Tukey double arcsine square-root transformation (FTT) [2].

The logit transformation [2, 3] is the log of the odds, that is, the log of the ratio of a proportion to its complement (one minus the proportion). By taking the natural logarithm of the odds, the logit transformation maps proportions onto the entire real number line. The logit transformed proportion (${\widehat{t}}_{i}$) with a continuity correction (*z*) for extreme proportions of zero or one is given by:

1 $${\widehat t}_i=\left\{\begin{array}{c}\text{ln}\left[\frac{e_i-z}{n_i}/(1-\frac{e_i-z}{n_i})\right]\ if\ e_i=n_i\\\text{ln}\left[\frac{e_i+z}{n_i}/(1-\frac{e_i+z}{n_i})\right]{ }\ if\ e_i=0\\\text{ln}\left[\frac{e_i}{n_i}/(1-\frac{e_i}{n_i})\right]\text{ }\ otherwise\end{array}\right.$$

where ${e}_{i}$ denotes the events and ${n}_{i}$ the sample size for each sample *i* = 1,2,3…*k* and for the two extreme situations (i.e. ${e}_{i}={n}_{i}, \ {e}_{i}=0$), a continuity correction (*z*) is applied [4], where *z* generally takes the value of 0.5. No continuity correction is applied to other event numbers.

The variance of the above transformed proportion (${\text{Var}}_{{\widehat{t}}_{i}}$) is given by:

2 $${\text{Var}}_{{\widehat t}_i}=\left\{\begin{array}{c}\frac1{{\text{e}}_\text{i}-z}\hspace{0.17em}+\hspace{0.17em}\frac1{{\text{n}}_\text{i}\hspace{0.17em}-\hspace{0.17em}{(\text{e}}_\text{i}-z)}\;\;if\;e_i=n_i\\\frac1{{\text{e}}_\text{i}+z}\hspace{0.17em}+\hspace{0.17em}\frac1{{\text{n}}_\text{i}\hspace{0.17em}-\hspace{0.17em}{(\text{e}}_\text{i}+z)}\;\;if\;e_i=0\\\frac1{{\text{e}}_\text{i}}\hspace{0.17em}+\hspace{0.17em}\frac1{{\text{n}}_\text{i}\hspace{0.17em}-\hspace{0.17em}{\text{e}}_\text{I}}\;\;\;\;\;\;\;otherwise\end{array}\right.$$

The double arcsine square root transformation [5] (FTT or the angular transformation) is calculated as two times the arcsine of the square root of the proportion. By applying the arcsine function twice, the transformed values are mapped from the interval $[0, 1]$ onto the interval $[0, \pi ]$. This transformation stabilizes the variance, mitigating the heteroscedasticity commonly observed in prevalence data. Also, the FTT promotes symmetrical distributions, enabling researchers to utilize parametric statistical tests that assume normality. It is therefore well suited to meta-analysis and the analysis of binomial or proportional data. The Freeman–Tukey double-arcsine transformed proportion (${t}_{i}$) is calculated as follows:

3 $${\widehat t}_i=\left\{\begin{array}{c}\text{arcsin}\sqrt{\frac{e_i-z}{n_i+1}}+arcsin\sqrt{\frac{e_i-z+1}{n_i+1}} \ \ if \ \ e_i=n_i\\\text{arcsin}\sqrt{\frac{e_i+z}{n_i+1}}+arcsin\sqrt{\frac{e_i+z+1}{n_i+1}} \ \ if \ \ e_i=0\\arcsin\sqrt{\frac{e_i}{n_i+1}}+arcsin\sqrt{\frac{e_i+1}{n_i+1}} \ \ otherwise\end{array}\right.$$

Again, ${n}_{i}$ denotes the sample size and ${e}_{i}$ denotes events for each sample *i* = 1,2,3…*k.* Although no continuity correction is required for this transformation, the benefit of adding a correction to events at the two extremes (${e}_{i}={n}_{i}, \ { e}_{i}=0$) was examined through simulation and found to be present and optimal at *z* = 0.15 which is different from the value found to be optimal for the logit transformation (see methods section). The variance of the above transformed proportion (${\text{Var}}_{{\widehat{t}}_{i}}$) is given by

4 $${\text{Var}}_{{\widehat{t}}_{i}} =\frac{1}{{n}_{i}+0.5}$$

The lower (${\widehat{t}}_{iL}$) and upper (${\widehat{t}}_{iH}$) confidence intervals of the transformed proportions (logit or FTT) are given by:

5 $${\widehat{t}}_{iL}={\widehat{t}}_{i}-1.96{\text{SE}}_{{\widehat{t}}_{i}}\text{ and }{\widehat{t}}_{iH}={\widehat{t}}_{i}+ 1.96{\text{SE}}_{{\widehat{t}}_{i}}$$

where ${\text{SE}}_{{\widehat{t}}_{i}}=\sqrt{{\text{Var}}_{{\widehat{t}}_{i}}}$. As observed in expression {4} above, the variance of the FTT proportion depends only on the sample size, making it a fixed value. However, the variance of the logit transformation depends additionally on the event counts, which are random variables [3] that vary from sample to sample, and this variability would contribute to the inconsistency of the logit transformation. As noted by Lin and Xu [3], this is potentially a great advantage of the FTT transformation, particularly in the meta-analysis context. However, recent research has raised two specific criticisms. Firstly, that the final transformation back to the proportion scale requires making a choice of estimate for the average sample size across studies. At the time when Miller [6], suggested a more accurate back-transformation for the FTT, it required a sample size, and he suggested the harmonic mean for meta-analytic estimates, but without solid justification. A recent criticism of FTT was based on use of the harmonic mean [7]. However, the latter ignored our much earlier demonstration that the effective sample size through the variance is more appropriate and restores stable estimation through the FTT [2, 8]. This improved approach has been implemented into the popular user-written meta-analysis module for Stata, **metan** [9]. The second, more recent, criticism that has emerged revolves around the inconsistency between the transformed values and the original proportions [10]. In a recently published article, the authors demonstrated that transformed values are not monotonic with the original proportions as they vary also with sample size (this is because of the “ + 1” in expression {3}) and they pointed out that FTT values differed by sample size even when the proportion value was held constant [10]. This led the authors to recommend *against* use of the FTT in meta-analysis [10]. Since the FTT has performed well in empirical studies [11], this criticism warrants further examination. The aim of the current study is to demonstrate, using simulation, the performance of FTT across a range of data-generating mechanisms, for both single and meta-analytic prevalence proportions, to identify the extent of potential non-monotonicity [10] and the consequences for inference of using the FTT. In so doing, we aim to determine whether FTT remains the better option, when compared to the logit transformation for meta-analysis of prevalence.

## Methods

### Simulations of single proportions to demonstrate their suitability as input into meta-analysis

We generated simulated datasets of proportions using Monte Carlo simulation in Stata. The proportions were then transformed using the FTT and logit transformations to evaluate variance stabilization as well as width and coverage of the confidence intervals prior to their input into meta-analysis. The simulation parameters were adjusted to evaluate the estimators under different scenarios, including extremely small proportions ranging from 0.0001 to 0.01, with sample sizes varying from 2500 to 12,500. Additionally, we examined the performance of the transformations for extremely large proportions ranging from 0.99 to 0.9999, again with varying sample sizes. Lastly, we evaluated non-extreme proportions ranging from 0.01 to 0.99, also with varying sample sizes ranging from 2500 to 12,500. We also assessed the optimal magnitude of *z* for the two extremes of events before settling on *z* = 0.5 and *z* = 0.15 for the logit and FTT transforms respectively (data not shown).

To further investigate the relative impact of sample size and proportion size on the variability of the FTT transformed proportion, we used the simulated data as generated above and conducted a regression analysis to predict FTT from sample size and proportion size. The proportion size was categorized into 500 quantiles and the sample size into 20 quantiles and then used as predictive categorical variables in linear regression. The marginal predictions from models with different ranges of the quantiles were plotted to visually analyze the contribution of these two factors to FTT prediction.

All simulations conducted follow the general steps given below (Stata code and datasets are available in supplementary material):

1. Creating local parameters for simulation
2. Defining the loop for the simulation
3. Creating the variables within the loop for simulation
  3.1. Transform the proportion as required.
  3.2. Estimate standard error for the generated proportion on its scale.
  3.3. Calculate confidence intervals for the proportion.
  3.4. Back transform the confidence intervals if required.
  3.5. Generate indicators for performance evaluation within the loop (width and confidence interval).
4. Complete the remaining loops and append data generated to file
5. Generating performance measures

### Step 1: creating starting values of local parameters for simulation

At the outset, we create local parameters for the simulation. These include the starting sample size (S), a confidence factor (C = 2), a continuity correction (*z* = 0.15 for FTT and *z* = 0.5 for logit transformations) and number of iterations (B).

### Step 2: defining the loop for the simulation

We start a loop defined by the true proportion (${p}_{t}$) that takes on a fixed value ranging between 0.0001 to 0.01 in steps of 0.00005 (199 values of ${p}_{t}$).

### Step 3: creating the variables within the loop for simulation

At each loop, we generate a set of B observations indexed by *i* = 1,2,3…B. For each observation the following variables are generated:

Sample size given by ${n}_{i}\sim int[Uniform\left(S,S+10000\right)]$ .

Events given by ${e}_{i}\sim Binomial({n}_{i},{p}_{t})$

The sample proportion is given by ${p}_{i}\hspace{0.17em}=\hspace{0.17em}\frac{{e}_{i}}{{n}_{i}}$

- Step 3.1. Transform the proportion as required: Each proportion is transformed using both the FTT and logit transforms as per the expressions {1} & {3}.
- Step 3.2. Estimate the standard error for the generated proportion on the respective scales as per expressions {2} & {4}.
- Step 3.3. Calculate confidence intervals for the proportion as per notation {5}.
- Step 3.4. Back-transformation of the transformed proportions, and estimation of lower and upper confidence intervals of the same.

When events are close to 0 or *n*_*i*_, the back transformed confidence limits for very small proportions can become numerically unstable and to avoid this our group had previously recommended [2] that the confidence interval of the back transformation be set to zero or 1 based on the value of a defined confidence variable *C* dropping below *C* = *2*. The back transformation of the logit proportion is given by:

6 $${\widehat{p}}_{i}= \frac{\text{exp}({\widehat{t}}_{i})}{\text{exp}\left({\widehat{t}}_{i}\right)+1}$$

The lower confidence intervals of the back-transformed logit proportion (${\widehat{p}}_{iL}$) is given by:

7 $${\widehat{p}}_{iL}= \left\{\begin{array}{cc}0& if\ {p}_{i}{n}_{i}\hspace{0.17em}<C\\ \frac{\text{exp}({\widehat{t}}_{iL})}{\text{exp}\left({\widehat{t}}_{iL}\right)+1}& if\ {p}_{i}{n}_{i}\hspace{0.17em}\ge C\end{array}\right.$$

The upper confidence intervals of the back-transformed logit proportion (${\widehat{p}}_{iH}$) is given by:

8 $${\widehat{p}}_{iH}= \left\{\begin{array}{cc}1& if\ {(1-p}_{i}){n}_{i}<C\\ \frac{\text{exp}({\widehat{t}}_{iH})}{\text{exp}\left({\widehat{t}}_{iH}\right)+1}& if\ {(1-p}_{i}){n}_{i}\ge C\end{array}\right.$$

where ${\widehat{t}}_{i},{\widehat{t}}_{iL},{\widehat{t}}_{iH}$ are the logit transformed proportions of interest and *C* is the confidence factor.

The back transformation of the FTT proportion is given by:

9 $${\widehat p}_i=\begin{array}{cc}0.5\;\left(1-sgn(\text{cos}{\widehat t}_i)\;\left[1-\left(\text{sin}{\widehat t}_i+\frac{\left(\text{sin}{\widehat t}_i-\frac1{\text{sin}{\widehat t}_i}\right)}{n_i}\right)^2\right]^{0.5}\right)&\end{array}$$

The lower confidence interval of the back-transformed FTT proportion (${\widehat{p}}_{iL}$) is given by:

10 $${\widehat p}_{iL}=\left\{\begin{array}{cc}0&if\;p_in_i<C\\0.5\left(1-sgn(\text{cos}{\widehat t}_{iL})\left[1-\left(\text{sin}{\widehat t}_{iL}+\frac{\left(\text{sin}{\widehat t}_{iL}-\frac1{\text{sin}{\widehat t}_{iL}}\right)}{n_i}\right)^2\right]^{0.5}\right)&if\;p_in_i\geq C\end{array}\right.$$

The upper confidence intervals of the back-transformed FTT proportion (${\widehat{p}}_{iH}$) is given by:

11 $${\widehat p}_{iH}=\left\{\begin{array}{cc}1&if\;(1-p_i)n_{i\;}<C\\0.5\left(1-sgn(\text{cos}{\widehat t}_{iH})\left[1-\left(\text{sin}{\widehat t}_{iH}+\frac{\left(\text{sin }t_i-\frac1{\text{sin }{\widehat t}_{iH}}\right)}{n_i}\right)^2\right]^{0.5}\right)&if\;(1-p_i)n_{i\;}\geq C\end{array}\right.$$

where ${\widehat{t}}_{i},{\widehat{t}}_{iL},{\widehat{t}}_{iH}$ are the FTT proportions of interest and *C* is the confidence factor.

- Step 3.5. Generate indices for performance evaluation within the loop.

We generate the following indicators to evaluate the performance of the estimators as detailed in the next step.

A. Width of confidence interval.
  The width of the CI$({W}_{i})$ for each loop is given by the difference between the upper and lower confidence interval corresponding to each iteration.
  $${W}_{i}=({\widehat{p}}_{iH}-{\widehat{p}}_{iL})$$
  where ${\widehat{p}}_{iH}$ is the upper CI and ${\widehat{p}}_{iL}$ is the lower CI for each iteration with $i=\text{1,2},3\dots \text{B}$
B. Coverage
  We generate a coverage indicator variable $({I}_{i})$ that returns 1 for coverage (i.e. if the confidence interval in the iteration set contains the true proportion) and 0 for no coverage.

### Step 4: Complete the remaining loops and append data generated to file

Run step 3 for each value of ${p}_{t}$. The output of each loop is appended and stored in a temporary file for generating the final output.

### Step 5: Generating Performance measures

We evaluate the performance of the estimator based on the average width of confidence interval and the coverage probability. A good estimator must have coverage probability close to its nominal level, and preferably with narrower confidence intervals. For the same reason, the coverage and width of the CI should be considered together while evaluating performance of the estimator.

The final average width at each run is given by

12 $$Width=\frac{\sum_{i=1}^{B}({\widehat{p}}_{iH}-{\widehat{p}}_{iL})}{B}$$

where $B$ is the no. of iterations in the simulation run, ${\widehat{p}}_{iH}$ is the upper CI and ${\widehat{p}}_{iL}$ is the lower CI for each iteration with $i=\text{1,2},3\dots \text{B}$

Coverage probability of an estimator indicates the proportion of intervals in the each run containing the true proportion. The sum of the coverage variable ${I}_{i}$ divided by the number of iterations for the simulation run returns the coverage probability:

13 $$\text{Pr}({I}_{i}=1)=\frac{\sum_{\text{i}=1}^{\text{B}}{I}_{i}}{\text{B}}$$

To visually analyze the results, we further plot the true proportion against the coverage probability.

### Simulation of application of the two transforms and back transforms to meta-analyses

We created three new sets of simulated datasets with the true proportions ranging from extremely small to extremely large proportions. The first set included true proportions between 0.0001 to 0.00105 in steps of 0.00005 (20 unique true proportions) and with 1000 iterations per true proportion creating 20,000 simulated proportions (based on events and observations). This simulation run was repeated nine more times to create a dataset with 10 runs each of 20 true proportion groups and 20,000 simulated proportions. From the dataset, groups of 10 proportions were randomly sampled from each true proportion group across each run to create the meta-analysis dataset and thus there were 20 meta-analysis datasets with 10 studies per run and 200 meta-analysis datasets in total. The same process was repeated to generate a dataset of 200 meta-analyses with extremely large true proportions ranging between 0.99892 to 0.9999 in steps of 0.00005 and another dataset of 200 meta-analyses for non-extreme proportions ranging from 0.1 to 0.87 in steps of 0.04. The meta-analytic results for each set of 10 studies were generated using the IVhet model [12]. Doi’s IVhet model has been shown in simulation studies to fix error estimation by addressing overdispersion when compared to the traditional fixed-effect model [13]. Both the simulations and the meta-analyses were computed in Stata version 17, StataCorp, College Station, TX, USA, using the **metan** module [9]. The analysis was done twice, once with either transform on the same dataset. The width of the confidence interval as well as the deviation of each meta-analytical point estimate from the true proportion value were plotted for each run and each transform as clustered box plots. Lastly, total coverage of the confidence intervals across the 200 meta-analyses were compared across the two transformations used and under the three different scenarios.

The back transformation of the FTT proportion in a single study is usually given by expression {9}. However in meta-analysis the harmonic mean of study sizes has been used [7] in place of *n*_*i*_ in expression {9} but this has been based on a misunderstanding which we have corrected as follows [2, 8]:

14 $$\overline p=\left\{\begin{array}{cc}\overline p'&if\ \frac{\overline p'}{\overline v}<2\;or\ \frac{1-\overline p'}{\overline v}<2\\0.5\left(1-sgn(\text{cos}\overline t)\left[1-\left(\text{sin}\overline t+\frac{\left(\text{sin}\overline t-\frac1{\text{sin}\overline t}\right)}{1/\overline v}\right)^2\right]^{0.5}\right)&if\ \frac{\overline p'}{\overline v}\geq2\;or\ \frac{1-\overline p'}{\overline v}\geq2\end{array}\right.$$

where $\overline{p }$ is the pooled prevalence on the natural scale and $\overline{t }$ is the pooled ${\widehat{t}}_{i}$. $\overline{p }{\prime}$ is given by $\overline{p }{^{\prime}}={\left(\text{sin}\frac{\overline{t}}{2 }\right)}^{2}$ and $\overline{v }$ is the pooled variance on the FTT scale. Instead of the harmonic mean as suggested by Miller [6], we use the inverse of the meta-analytic variance on the double arcsine transformed scale ($1/\overline{v }$) which represents the effective meta-analytic sample size. Previous studies reported by our group suggest that this is a more realistic estimate than the harmonic mean [2].

The lower confidence intervals of the back-transformed pooled proportion (${\overline{p} }_{iL}$) is given by:

15 $${\overline{p} }_{iL}= \left\{\begin{array}{cc}0& if\ \frac{\overline{p }{\prime}}{\overline{v} }<2\\ 0.5 \left(1-sgn (\text{cos}\overline{\overline{t}}) {\left[1-{\left(\text{sin}\overline{\overline{t}}+\frac{\left(\text{sin}\overline{\overline{t}}-\frac{1}{\text{sin}\overline{\overline{t}}}\right)}{1/\overline{v} }\right)}^{2} \right]}^{0.5} \right)& if\ \frac{\overline{p }{\prime}}{\overline{v}}\ge 2\end{array}\right.$$

and the upper confidence intervals of the back-transformed pooled proportion (${\widehat{p}}_{iH}$) is given by:

16 $${\overline{p} }_{iH}= \left\{\begin{array}{cc}1& if\frac{1-\overline{p }{\prime}}{\overline{v} }<2\\ 0.5 \left(1-sgn (\text{cos}\overline{\overline{t}}) {\left[1-{\left(\text{sin}\overline{\overline{t}}+\frac{\left(\text{sin}\overline{\overline{t}}-\frac{1}{\text{sin}\overline{\overline{t}}}\right)}{1/\overline{v} }\right)}^{2} \right]}^{0.5} \right)& if\frac{1-\overline{p }{\prime}}{\overline{v}}\ge 2\end{array}\right.$$

where $\overline{\overline{t}}=\overline{t}\pm {Z }_{\alpha /2}\sqrt{\overline{v} }$. This procedure has been implemented in the **metan** module of Stata which was used for this analysis.

## Results

### Coverage of single proportions

**Fig. 1:**
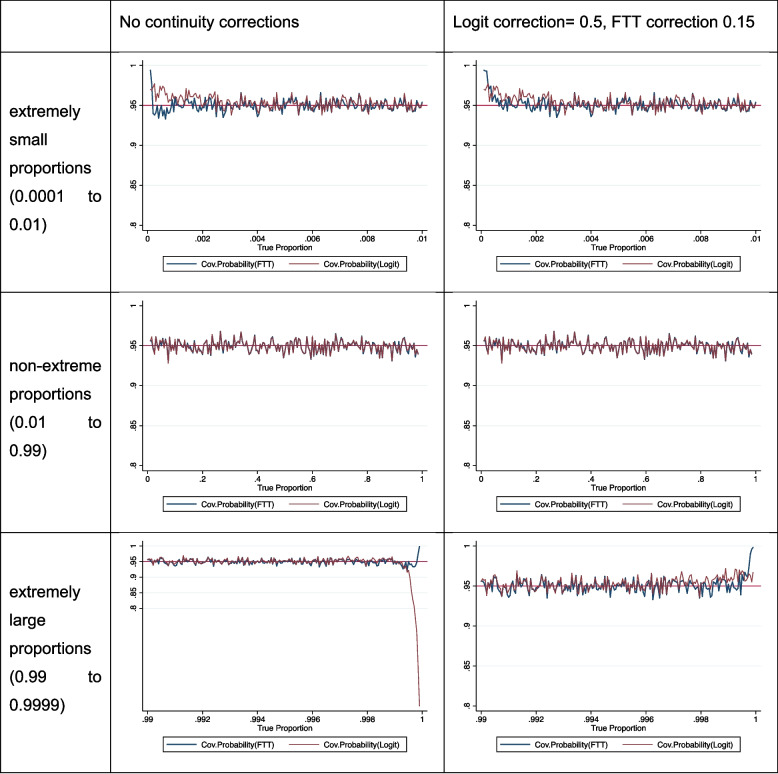
Coverage probability under both transformations (single proportions)

In the first part of our results, we compared the coverage of the FTT and logit transformed proportions at the extremes of magnitude of the proportions (Fig. 1). For extremely large proportions, the logit transformation has grossly inadequate coverage at values close to 1. For extremely small proportions and non-extreme proportions, both logit and FTT have similar coverage.

We then applied corrections to adjust for events equal to zero or events equal to the sample size in the simulated datasets. The corrected event count are given in expressions {2} and {4} and we observe that by using corrections of 0.15 for the FTT and 0.5 for the logit transformation, both estimators achieved optimal coverage at both ends of extreme proportions (Fig. 1). These are helpful because at these extremes of proportions (0 and 1) the transform values also become very extreme and these corrections help mitigate this. For non-extreme proportions, both the FTT and logit transformations yielded similar coverage across all scenarios.

### Width of the back-transformed CI for single proportions

**Fig. 2:**
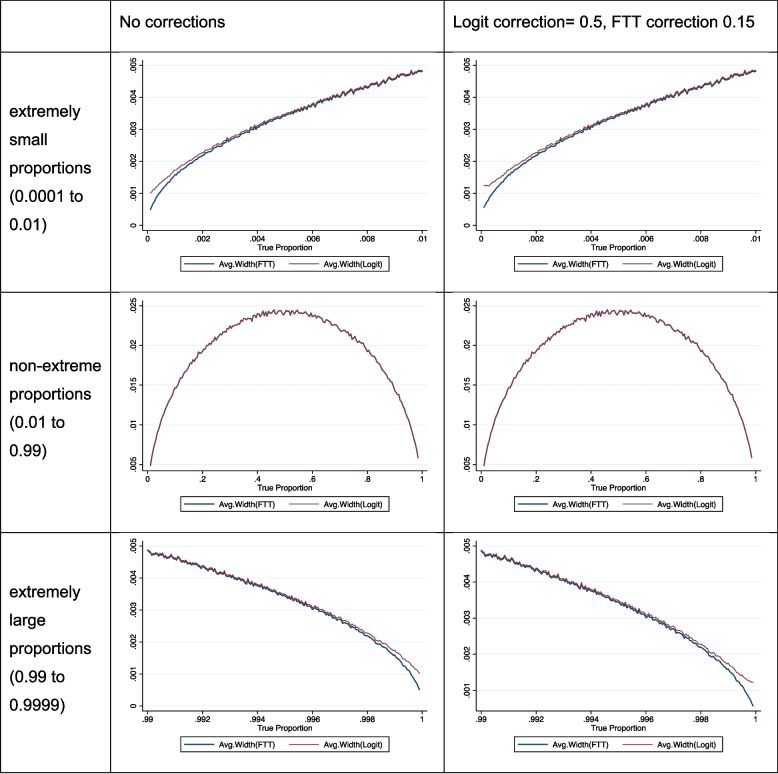
Width of the back-transformed CI under both transformations (single proportions)

The subsequent analysis aimed to compare the mean width of the back-transformed confidence intervals (CI) obtained using both the transformations (Fig. 2). The FTT exhibited a smaller width of the CI compared to the logit transformation at both extremely small and extremely large proportions. The width of the confidence intervals remained the same with or without the continuity corrections employed (0.15 for the FTT and 0.5 for the logit transformation).

### Variability of the FTT with sample size

**Fig. 3:**
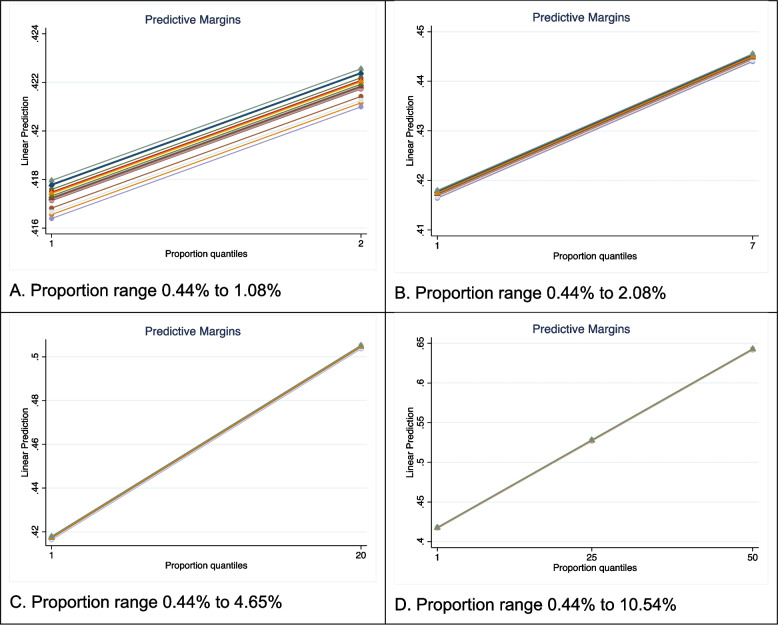
Variability of FTT with sample size quantiles (different color lines) for single proportions

The regression analysis showed that the highest variability of the FTT occurred when the range of proportion was limited to quantiles 1 – 2 resulting in a very small range in proportions from 0.0044 to 0.0108 (Fig. 3, Panel A). Within this narrow range, the FTT varied by sample size quantile in a very miniscule range from 0.416 to 0.423. As the range of proportion increased (quantiles 1 – 7; proportions from 0.0044 to 0.0208), we observed a decrease in the observed variability by sample size quantile as proportion changed the FTT much more compared to sample size (Fig. 3, Panel B). When the range of proportions was increased to include quantiles 1 – 20 (range of proportions 0.0044 to 0.0465) there was barely any significant variation by sample size quantile (Fig. 3, Panel C), and finally when quantiles 1 – 50 were included (range of proportions 0.0044 to 0.1054), no variability due to sample size quantile was discernable (Fig. 3, Panel D), indicating that it was not relevant in relation to the changes due to the size of the proportion.

### Performance in meta-analyses

**Fig. 4:**
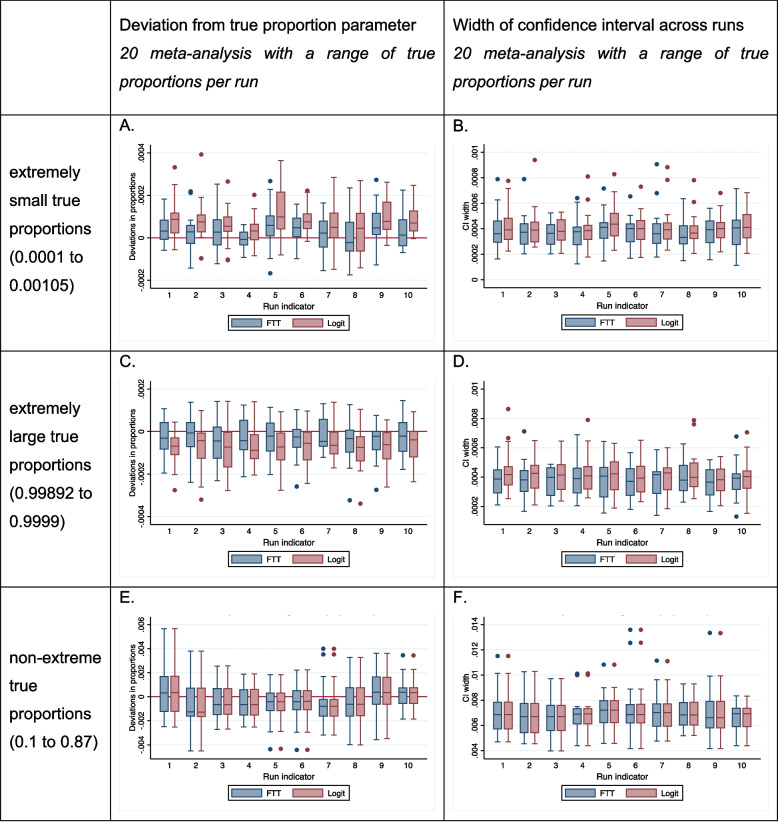
Performance of both transformations (meta-analysis)

We analyzed and compiled results from the three sets of simulated meta-analyses generated which were for extremely small proportions, extremely large proportions and non-extreme proportions as explained earlier. For both extremely small and large proportions, the FTT based meta-analyses exhibited notably lesser relative deviation from the true proportion in comparison to the logit transformation based meta-analyses (as depicted in Fig. 4, panels A & C). The FTT consistently yielded narrower confidence intervals when compared to the logit transformation in both the scenarios, as indicated in Fig. 4, panels B & D. For extremely small proportions, the FTT demonstrated decreased width with a coverage probability (94.5%) close to the nominal level across simulation runs, surpassing the logit transformation which had 86% coverage. Similarly, for extremely large proportions, the FTT demonstrated decreased width of the confidence interval and coverage probability at the nominal level (95%), compared to the logit transformation which had 87.5% coverage. When analyzing non-extreme proportions, both FTT and logit transformed proportions demonstrated similar performance (Fig. 4) with no marked difference in deviation from the true proportion or in coverage of the confidence interval (95% with both transforms). All three meta-analytic simulated datasets are attached in the supplementary material. We should emphasize here that non-monotonicity of FTT values is always seen when sample sizes differ and the proportion is held constant but this does not really matter because the impact of the proportion on the FTT value far exceeds that of changes in sample size as can be inferred from the results above.

### Application to a real world meta-analysis

**Fig. 5:**
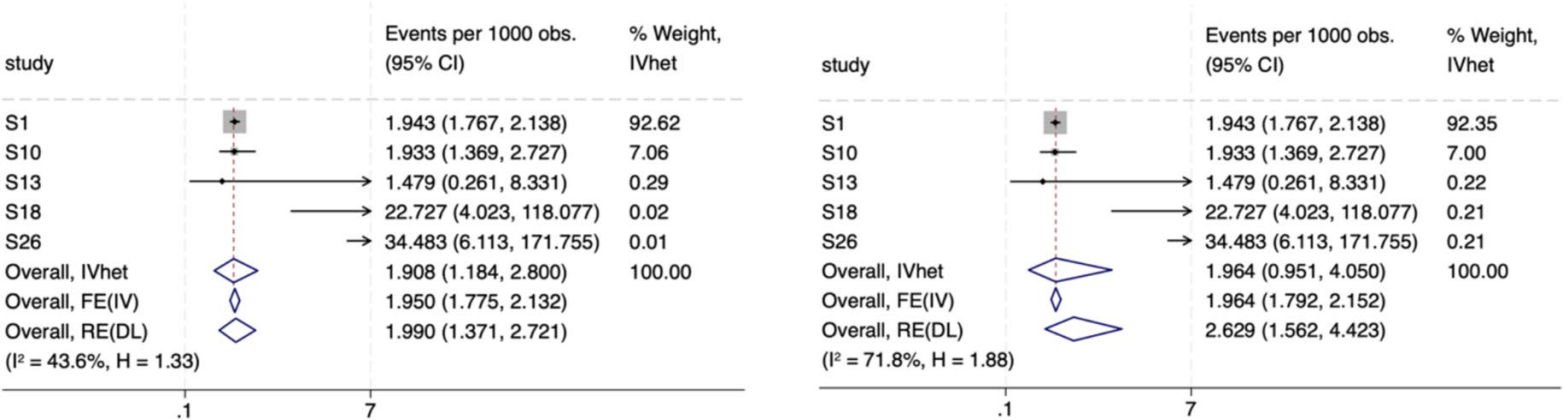
Forest plots of hepatitis C virus (HCV) meta-analysis with Freeman-Tukey transformation (left) and logit transformation (right) and utilizing three synthesis models (IVhet, FE (IV estimator) and RE (DL estimator). *IVhet, inverse variance heterogeneity model; FE (IV), fixed effect model with inverse variance estimator; RE(DL), random effects model with DerSimonian-Laird estimator

To illustrate the practical implications of our findings, we applied both the Freeman-Tukey (FTT) and logit transformations to a meta-analysis estimating the prevalence of hepatitis C virus (HCV) infections in the general population of Nepal. This analysis includes a subset of five studies from an unpublished dataset containing 28 studies [14]. The meta-analysis pooled proportions ranging from 0.001 to 0.034, reflecting the low prevalence of hepatitis C virus (HCV) infections in the studied population. Figure 5 presents the results, with the left panel using the FTT transformation and the right panel using the logit transformation.

The comparison between the two transformations highlights key differences. The FTT transformation results in a pooled prevalence estimate of 1.908 per 1000 observations (95% CI: 1.184 – 2.800) using the IVhet model, while the logit transformation produces a higher pooled estimate of 1.964 per 1000 (95% CI: 0.951 – 4.050) with increased variability. The logit transformation also shows higher heterogeneity (I^2^ = 71.8%, H = 1.88) compared to FTT (I^2^ = 43.6%, H = 1.33), indicating that the logit transformation leads to less stable estimates and wider confidence intervals, particularly for studies with extreme proportions.

Further, comparing meta-analysis models within the FTT-transformed dataset (left panel), we observe that the choice of model matters and both the inverse variance (IV) and DerSimonian-Laird (DL) random-effects models demonstrate differences in point estimation as well as precision. Even within transformation choice, the three models result in different point estimates and/or precision Given that some degree of heterogeneity can occur by chance, selecting a model that properly handles overdispersion is essential. Since fixed-effect models do not have a mechanism for this, we focused on models that do, ultimately selecting IVhet based on the simulation results. This practical example corroborates our simulation findings that FTT compared to logit transformations are not equivalent and that on top of this choices about models to use for such synthesis matters too.

## Discussion

### Overall findings

This study compared the performance of the FTT with the logit transformation and the findings collectively suggest that the FTT offers superior performance in terms of width of the confidence interval and coverage with both single proportions and meta-analyses. In addition, the FTT provided less deviation of the meta-analytic estimate from the true parameter indicating its potential as the preferred method for application in meta-analysis.

We observed that by applying a correction to event numbers equal to either zero or ${n}_{i}$ of 0.15 (FTT) and 0.5 (logit), the best coverage was obtained. While not directly an aim of this study, this is an important observation because it helps improve estimation. These corrections can be used as standard because they have no impact when the events observed are not zero or not equal to the sample size. Additionally, the FTT generally exhibited a smaller mean width of the CI compared to the logit transformation. The findings in this study therefore indicate that the FTT provides better coverage and narrower intervals for estimating proportions of an extreme size. Both transformations are generally robust and provide comparable results in scenarios of non-extreme proportions. The FTT however remains the better choice with extreme prevalence proportions. For non-extreme proportions, researchers can choose between the FTT and logit transformations based on their preferences and familiarity with the methods.

### Implications for researchers

The debate surrounding the appropriate methodology for meta-analysis of prevalence had been the subject of recent attention, initially sparked by the work by Schwarzer et al. [7]. Miller's original recommendation [6] has been found to lack sufficient supporting evidence and is likely flawed; however, this aspect seems to be underappreciated in the field, especially considering that Schwarzer et al. based their critique on Miller's approach. Prior to Schwarzer’s criticism, we had already published a suggested modification to Miller's proposal, which addresses these flaws and resolves the concerns raised [2, 8], but this contribution appears to have been overlooked. The FTT, in reality, yields robust results when used in two-step meta-analysis approaches, the latter also having the added benefit of retaining the study weights and allowing choice of more robust meta-analysis models, such as the IVhet model [8]. Another paper by Röver et al. also entered the discussion, highlighting a 'paradoxical behavior' in which FTT values differed by sample size, even when the proportion value remained constant [10]. Our findings, however, demonstrate that this issue does not translate into a problem for the use of FTT. The reason why this is not an issue is that the variability in FTT with even large variations in sample size is only equivalent to changes in a range of proportion sizes that are extremely small (~ 0.5% or 0.005) and the fluctuation in FTT values was also in a minuscule range of 0.416 to 0.423 with sample size. Thus, the FTT varies in the third decimal place (0.007), a reason why it has little impact on meta-analysis results. These findings emphasize that while there might be FTT variability with sample size, the range of the proportion takes precedence and the variability of the FTT with sample size is miniscule compared to its variation with size of the proportion which explains why we can demonstrate no impact in practice..

In addition, in meta-analysis, the FTT consistently shows a lower relative deviation from the true proportion parameter, producing narrower confidence intervals and achieving better coverage than the logit transformation, both when the proportions are extremely small or large. These findings collectively highlight the potential advantages associated with utilizing the FTT over the logit as the transformation of choice for meta-analysis of prevalence. This underscores the significance of methodological choice in ensuring robust and accurate outcomes in the meta-analysis of prevalence.

### Implications for practice

We provide evidence why the FTT is a strong choice of transformation in meta-analysis of prevalence, especially when proportions are extreme, outperforming the logit transformation in our simulation. The comparison between FTT and logit transformations in a real-world meta-analysis of hepatitis C virus (HCV) prevalence in Nepal (Fig. 5) highlights the superior performance of FTT, yielding narrower confidence intervals, lower heterogeneity, and more stable pooled estimates. The logit transformation, in contrast, exhibits higher variability and wider confidence intervals, particularly for studies with very low or high prevalence estimates. Additionally, our results emphasize that meta-analysis model selection further influences estimation accuracy. The IVhet model, when paired with FTT, provides the most robust pooled estimate, effectively handling overdispersion without inflating variance like the DerSimonian-Laird random-effects model. The simulations also provide empirical evidence that even though the sample size alters the FTT value in addition to the proportion size, the sample size effect is so small that it does not impact the other benefits of this transform, and it still retains better performance for meta-analysis of prevalence.

The methods we discuss in this paper are all available via the **metan **module in Stata. This module can be downloaded and installed by typing **ssc install metan **into the command window in Stata. The particular form of the FTT back transformation using the inverse of the meta-analytic variance which is discussed in this paper, is also available through this module using the option: **tr(ftukey, iv).**

### Strengths and potential limitations

We should acknowledge that no single transformation is universally optimal, and researchers should carefully consider the characteristics of their dataset when selecting an appropriate method. We also caution that this study employed simulated datasets and while our findings provide valuable insights, it is essential to consider the specific characteristics of the dataset under analysis. Different datasets may exhibit unique patterns, and researchers should carefully assess the suitability of the transformation selected based on their specific data properties.

### Conclusion

The FTT seems to be the transformation of choice when contemplating aggregate data meta-analysis of prevalence proportions. The logit transformation works just as well when proportions are not extreme and is a suitable alternative in this situation. If the FTT transformation is chosen, care should be exercised regarding the form of back transformation utilized and we recommend use of inverse variance in lieu of mean sample size options when converting FTT back to natural proportions.

## Supplementary Information

Supplementary Material 1

## Data Availability

Stata codes for the generation of simulated datasets are provided as supplementary material.

## Abbreviations

FTT: Freeman–Tukey double arcsine square-root transformation
CI: Confidence interval

## References

[1] Fleiss JL, Levin B, Paik MC. Statistical Methods for Rates and Proportions, Wiley Series in Probability and Statistics, 1940-6347. Wiley; 2003. 10.1002/0471445428.

[2] Barendregt JJ, Doi SA, Lee YY, Norman RE, Vos T. Meta-analysis of prevalence. J Epidemiol Community Health. 2013;67:974–8. 23963506 10.1136/jech-2013-203104

[3] Lin L, Xu C. Arcsine-based transformations for meta-analysis of proportions: Pros, cons, and alternatives. Health Sci Rep. 2020;3: e178. 32728636 10.1002/hsr2.178 PMC7384291

[4] Hautus MJ. Corrections for extreme proportions and their biasing effects on estimated values ofd′. Behav Res Methods Instrum Comput. 1995;27:46–51.

[5] Freeman MF, Tukey JW. Transformations related to the angular and the square root. Ann Math Statist. 1950;21:607–11.

[6] Miller JJ. The Inverse of the Freeman – Tukey Double Arcsine Transformation. Am Stat. 1978;32:138–138.

[7] Schwarzer G, Chemaitelly H, Abu-Raddad LJ, Rücker G. Seriously misleading results using inverse of Freeman-Tukey double arcsine transformation in meta-analysis of single proportions. Res Synth Methods. 2019;10:476–83. 30945438 10.1002/jrsm.1348 PMC6767151

[8] Doi SA, Xu C. The Freeman-Tukey double arcsine transformation for the meta-analysis of proportions: Recent criticisms were seriously misleading. J Evid Based Med. 2021;14:259–61. 34632718 10.1111/jebm.12445

[9] Fisher D, Harris R, Bradburn M, Deeks J, Harbord R, Altman D, et al. "METAN: Stata module for fixed and random effects meta-analysis," Statistical Software Components S456798, Boston College Department of Economics. 2006. Revised 15 July 2024. https://ideas.repec.org/c/boc/bocode/s456798.html. Accessed 14 Mar 2025.

[10] Röver C, Friede T. Double arcsine transform not appropriate for meta-analysis. Res Synth Methods. 2022;13:645–8. 35837800 10.1002/jrsm.1591

[11] Brown LD, Cai TT, DasGupta A. Interval Estimation for a Binomial Proportion. Statist Sci. 2001;16:101–33.

[12] Doi SAR, Barendregt JJ, Khan S, Thalib L, Williams GM. Advances in the meta-analysis of heterogeneous clinical trials I: The inverse variance heterogeneity model. Contemp Clin Trials. 2015;45:130–8. 26003435 10.1016/j.cct.2015.05.009

[13] Doi SAR, Furuya-Kanamori L. Selecting the best meta-analytic estimator for evidence-based practice: a simulation study. Int J Evid Based Healthc. 2020;18:86–94. 31764215 10.1097/XEB.0000000000000207

[14] Naveira MCM, Badal K, Dhakal J, Mayer NA, Pokharel B, Del Prado RF. Seroprevalence of hepatitis B and C in Nepal: a systematic review (1973–2017). Hepatology Medicine Policy. 2018;3:10. 30288333 10.1186/s41124-018-0039-2 PMC6126038

