# Supplementary material for: Overcoming challenges in prevalence meta-analysis: the case for the Freeman-Tukey transform
Source: BMC Med Res Methodol. 2025 Apr 5;25:89. doi: 10.1186/s12874-025-02527-z (PMC11971753; doi:10.1186/s12874-025-02527-z)
Supplement: Supplementary file 1 — Supplementary Material 1 [file 12874_2025_2527_MOESM1_ESM.docx]

***Supplementary material***

Stata codes for generating the simulated data and performing the analysis.

1. The below code was used for simulation data generation and to compare the performance (coverage and width of CI) of the FTT and logit transformed proportions under three scenarios- extremely small proportions ranging from 0.0001 to 0.01, extremely large proportions ranging from 0.99 to 0.9999, and non-extreme proportions ranging from 0.1 to 0.9.

clear all

local iter 1000

local samplesize 2500

local correction 2

local lt 0.5

local ft 0.15

set seed 1234

tempfile building

save `building', emptyok

****select range of true proportions from below three

forvalues y = .0001(.00005)0.01 {

***forvalues y = .99(.00005)0.9999 {

***forvalues y = .01(.005)0.99 {

clear

set obs `iter'

gen iterations = `iter'

gen trueprop = `y'

gen n = (runiform(`samplesize',`samplesize'+10000))

replace n=round(n)

gen factor = `correction'

gen cases = rbinomial(n,trueprop)

gen prop = cases/n

***FTT***

gen casesf=cases

replace casesf= cases-`ft' if cases==n

replace casesf = cases+`ft' if cases==0

gen ftt = asin(sqrt(casesf / (n + 1))) + asin(sqrt((casesf + 1)/(n + 1)))

gen seftt = sqrt(1/(n + 0.5))

gen lciftt = ftt - (1.96*seftt)

gen hciftt = ftt + (1.96*seftt)

gen lciprop = .

gen lnum=.

replace lciprop = (((0.5*(1-((sign(cos(lciftt)))*((1-((sin(lciftt)+(((sin(lciftt))-(1/sin(lciftt)))/n))^2))^0.5))))))

replace lciprop = 0 if (prop/(seftt*seftt)) < factor

replace lnum = 1 if (prop/(seftt*seftt)) < factor

gen hciprop = .

gen hnum=.

replace hciprop = (((0.5*(1-((sign(cos(hciftt)))*((1-((sin(hciftt)+(((sin(hciftt))-(1/sin(hciftt)))/n))^2))^0.5))))))

replace hciprop = 1 if ((1-prop)/(seftt*seftt)) < factor

replace hnum = 1 if ((1-prop)/(seftt*seftt)) < factor

gen coverf = .

replace coverf = 1 if trueprop>=lciprop & trueprop<=hciprop

replace coverf = 0 if trueprop<lciprop | trueprop>hciprop

egen sumcoverf = total(coverf)

gen coverprobf = sumcover/iterations

gen widthf = hciprop-lciprop

qui summ widthf, det

gen avwidthf = r(mean)

****logit****

gen casesl=cases

replace casesl= cases-`lt' if cases==n

replace casesl = cases+`lt' if cases==0

gen lgprop = logit(casesl/n)

gen selgprop = sqrt((1/casesl) + (1/(n-casesl)))

gen lcilgprop = lgprop - (1.96*selgprop)

gen hcilgprop = lgprop + (1.96*selgprop)

gen lcipropl = .

replace lcipropl = invlogit(lcilgprop)

replace lcipropl = 0 if (prop*n) < factor

gen hcipropl = .

replace hcipropl = invlogit(hcilgprop)

replace hcipropl = 1 if ((1-prop)*n) < factor

gen coverl = .

replace coverl = 1 if trueprop>=lcipropl & trueprop<=hcipropl

replace coverl = 0 if trueprop<lcipropl | trueprop>hcipropl

egen sumcoverl = total(coverl)

gen coverprobl = sumcoverl/iterations

gen widthl = hcipropl-lcipropl

qui summ widthl, det

gen avwidthl = r(mean)

keep iterations trueprop factor coverprobf coverprobl cases n prop coverf coverl lciprop hciprop ftt seftt avwidthf lnum hnum lcipropl hcipropl lgprop selgprop avwidthl

**** Append output for analysis

append using `building'

save `"`building'"', replace

}

egen propid = group(trueprop)

order propid, first

bysort propid: gen iterid=_n

order iterid, after(propid)

replace coverprobf=. if iterid !=1

replace avwidthf =. if iterid !=1

replace coverprobl=. if iterid !=1

replace avwidthl =. if iterid !=1

label var coverprobf "Cov.Probability(FTT)"

label var coverprobl "Cov.Probability(Logit)"

label var trueprop "True Proportion"

label var avwidthf "Avg.Width(FTT)"

label var avwidthl "Avg.Width(Logit)"

****coverage graph (combined)

line coverprobf coverprobl trueprop , yline(0.95) yla(0.8 0.85 0.9 0.95 1) lwidth(medthick) graphregion(color(white)) bgcolor(white)

****confidence interval width graph (combined)

line avwidthf avwidthl trueprop , lwidth(medthick) graphregion(color(white)) bgcolor(white)

1. Use the below codes to demonstrate the variability of the FTT transform with sample size and proportion size. *Use the non-extreme dataset from above*

***** FTT variability

xtile propcat=prop,nq(500)

xtile ncat=n,nq(20)

mkspline propcats=propcat,cubic nk(3)

regress ftt propcats* i.ncat

linktest

label var propcats1 "Proportion quantiles"

*** margin plots choose one set

margins, over(ncat) at(propcats1=(1 2))

marginsplot, sch(s2color) legend(off) noci graphregion(fcolor(white))

margins, over(ncat) at(propcats1=(1(1)7))

marginsplot, sch(s2color) legend(off) noci graphregion(fcolor(white))

margins, over(ncat) at(propcats1=(1(1)20))

marginsplot, sch(s2color) legend(off) noci graphregion(fcolor(white))

margins, over(ncat) at(propcats1=(1(5)50))

marginsplot, sch(s2color) legend(off) noci graphregion(fcolor(white))

1. The code given below was used for simulation data generation and comparing performance of the FTT and logit transformed proportions in meta-analysis of prevalence.

clear all

local iter 1000

local samplesize 2500

local correction 2

local lt 0.5

local ft 0.15

set seed 123451

tempfile building

save `building', emptyok

forvalues run= 1/10 {

***select one of the three ranges below

forvalues y = .99892(.00005)0.9999 {

***forvalues y = .0001(.00005)0.001051 {

***forvalues y = 0.1 (0.04) 0.87 {

clear

set obs `iter'

gen iterations = `iter'

gen trueprop = `y'

**gen n = (runiform(2/trueprop,(2/trueprop)+1000))

gen n = (runiform(`samplesize',`samplesize'+10000))

replace n=round(n)

gen factor = `correction'

gen cases = rbinomial(n,trueprop)

gen prop = cases/n

***FTT***

gen casesf=cases

replace casesf= cases-`ft' if cases==n

replace casesf = cases+`ft' if cases==0

gen ftt = asin(sqrt(casesf / (n + 1))) + asin(sqrt((casesf + 1)/(n + 1)))

gen seftt = sqrt(1/(n + 0.5))

gen lciftt = ftt - (1.96*seftt)

gen hciftt = ftt + (1.96*seftt)

gen lciprop = .

gen lnum=.

replace lciprop = (((0.5*(1-((sign(cos(lciftt)))*((1-((sin(lciftt)+(((sin(lciftt))-(1/sin(lciftt)))/n))^2))^0.5))))))

replace lciprop = 0 if (prop/(seftt*seftt)) < factor

replace lnum = 1 if (prop/(seftt*seftt)) < factor

gen hciprop = .

gen hnum=.

replace hciprop = (((0.5*(1-((sign(cos(hciftt)))*((1-((sin(hciftt)+(((sin(hciftt))-(1/sin(hciftt)))/n))^2))^0.5))))))

replace hciprop = 1 if ((1-prop)/(seftt*seftt)) < factor

replace hnum = 1 if ((1-prop)/(seftt*seftt)) < factor

gen coverf = .

replace coverf = 1 if trueprop>=lciprop & trueprop<=hciprop

replace coverf = 0 if trueprop<lciprop | trueprop>hciprop

egen sumcoverf = total(coverf)

gen coverprobf = sumcover/iterations

gen widthf = hciprop-lciprop

qui summ widthf, det

gen avwidthf = r(mean)

****logit****

gen casesl=cases

replace casesl= cases-`lt' if cases==n

replace casesl = cases+`lt' if cases==0

gen lgprop = logit(casesl/n)

gen selgprop = sqrt((1/casesl) + (1/(n-casesl)))

gen lcilgprop = lgprop - (1.96*selgprop)

gen hcilgprop = lgprop + (1.96*selgprop)

gen lcipropl = .

replace lcipropl = invlogit(lcilgprop)

replace lcipropl = 0 if (prop*n) < factor

gen hcipropl = .

replace hcipropl = invlogit(hcilgprop)

replace hcipropl = 1 if ((1-prop)*n) < factor

gen coverl = .

replace coverl = 1 if trueprop>=lcipropl & trueprop<=hcipropl

replace coverl = 0 if trueprop<lcipropl | trueprop>hcipropl

egen sumcoverl = total(coverl)

gen coverprobl = sumcoverl/iterations

gen widthl = hcipropl-lcipropl

qui summ widthl, det

gen avwidthl = r(mean)

keep iterations trueprop factor coverprobf coverprobl cases n prop coverf coverl lciprop hciprop ftt seftt avwidthf lnum hnum lcipropl hcipropl lgprop selgprop avwidthl

gen run = `run'

*****Append output for analysis

append using `building'

save `"`building'"', replace

}

}

egen propid = group(run trueprop)

order propid, first

order run, before(propid)

bysort propid: gen iterid=_n

order iterid, after(propid)

****Select 10 studies randomly

sample 10, by(propid) count

gen ma_es_ftt=.

gen ma_se_ftt=.

***Run meta-analyses

forvalues w = 1/200 {

metan cases n if propid== `w' , ivhet pr transform(ftukey, iv) nograph

replace ma_es_ftt = r(eff) if propid== `w'

replace ma_se_ftt = r(se_eff) if propid== `w'

}

gen ma_es_logit=.

gen ma_se_logit=.

forvalues w = 1/200 {

metan cases n if propid== `w' , ivhet pr transform(logit) nograph

replace ma_es_logit = r(eff) if propid== `w'

replace ma_se_logit = r(se_eff) if propid== `w'

}

***Backtransform results

bysort propid: gen study_id =_n

replace ma_es_ftt=. if study_id >1

replace ma_se_ftt=. if study_id >1

replace ma_es_logit=. if study_id >1

replace ma_se_logit=. if study_id >1

gen n_ma = 1/(ma_se_ftt^2)

gen ma_es_ftt_p = (((0.5*(1-((sign(cos(ma_es_ftt)))*((1-((sin(ma_es_ftt)+(((sin(ma_es_ftt))-(1/sin(ma_es_ftt)))/n_ma))^2))^0.5))))))

gen pn = ma_es_ftt_p*n_ma

gen revpn = (1-ma_es_ftt_p)*n_ma

replace ma_es_ftt_p = sin(ma_es_ftt/2)^2 if pn <2

replace ma_es_ftt_p = sin(ma_es_ftt/2)^2 if revpn <2

gen ma_lci_ftt= ma_es_ftt-(ma_se_ftt *1.96)

gen ma_lci_ftt_p = (((0.5*(1-((sign(cos(ma_lci_ftt)))*((1-((sin(ma_lci_ftt)+(((sin(ma_lci_ftt))-(1/sin(ma_lci_ftt)))/n_ma))^2))^0.5))))))

replace ma_lci_ftt_p = 0 if pn < factor

gen ma_uci_ftt= ma_es_ftt+(ma_se_ftt *1.96)

gen ma_uci_ftt_p = (((0.5*(1-((sign(cos(ma_uci_ftt)))*((1-((sin(ma_uci_ftt)+(((sin(ma_uci_ftt))-(1/sin(ma_lci_ftt)))/n_ma))^2))^0.5))))))

replace ma_uci_ftt_p = 1 if revpn < factor

gen ma_es_logit_p = invlogit(ma_es_logit)

gen ma_lci_logit_p= invlogit(ma_es_logit-(ma_se_logit *1.96))

replace ma_lci_logit_p = 0 if pn < factor

gen ma_uci_logit_p= invlogit(ma_es_logit+(ma_se_logit *1.96))

replace ma_uci_logit_p = 1 if revpn < factor

****Graph variables **

gen logitdif = ma_es_logit_p-trueprop

gen fttdif = ma_es_ftt_p-trueprop

gen zero = 0

gen logitcidif = ma_uci_logit_p - ma_lci_logit_p

gen fttcidif = ma_uci_ftt_p - ma_lci_ftt_p

label var logitdif "Logit"

label var fttdif "FTT"

label var logitcidif "Logit"

label var fttcidif "FTT"

***Deviation graph

graph box fttdif logitdif, over(run) b1title("Run indicator") ytitle("Deviations in proportions") title("Deviations from the true population parameter") subtitle("20 meta-analyses with a range of true proportions per run") yline(0) sch(s2color) graphregion(fcolor(white))

***CI width graph

graph box fttcidif logitcidif, over(run) b1title("Run indicator") ytitle("CI width") title("CI width across runs") subtitle("20 meta-analyses with a range of true proportions per run") sch(s2color) graphregion(fcolor(white))

***coverage probability

gen coverlogit = 1 if (trueprop >= ma_lci_logit_p) & (trueprop<= ma_uci_logit_p)

replace coverlogit = 0 if coverlogit==. & study_id==1

gen coverftt = 1 if (trueprop >= ma_lci_ftt_p) & (trueprop<= ma_uci_ftt_p)

replace coverftt = 0 if coverftt==. & study_id==1

summ coverftt coverlogit
